# Mesodiencephalic Dopaminergic Neuronal Differentiation Does Not Involve GLI2A-Mediated SHH-Signaling and Is under the Direct Influence of Canonical WNT Signaling

**DOI:** 10.1371/journal.pone.0097926

**Published:** 2014-05-27

**Authors:** Simone Mesman, Lars von Oerthel, Marten P. Smidt

**Affiliations:** Swammerdam Institute for Life Sciences, CNS, FNWI University of Amsterdam, Amsterdam, The Netherlands; School of Medicine and Health Sciences, University of North Dakota, United States of America

## Abstract

Sonic Hedgehog (SHH) and WNT proteins are key regulators in many developmental processes, like embryonic patterning and brain development. In the brain, SHH is expressed in a gradient starting in the floor plate (FP) progressing ventrally in the midbrain, where it is thought to be involved in the development and specification of mesodiencephalic dopaminergic (mdDA) neurons. GLI2A-mediated SHH-signaling induces the expression of *Gli1*, which is inhibited when cells start expressing SHH themselves. To determine whether mdDA neurons receive GLI2A-mediated SHH-signaling during differentiation, we used a BAC-transgenic mouse model expressing eGFP under the control of the *Gli1* promoter. This mouse-model allowed for mapping of GLI2A-mediated SHH-signaling temporal and spatial in the mouse midbrain. Since mdDA neurons are born from E10.5, peaking at E11.0–E12.0, we examined *Gli1*-eGFP embryos at E11.5, E12.5, and E13.5, indicating whether *Gli1* was induced before or during mdDA development and differentiation. Our data indicate that GLI2A-mediated SHH-signaling is not involved in mdDA neuronal differentiation. However, it appears to be involved in the differentiation of neurons which make up a subset of the red nucleus (RN). In order to detect whether mdDA neuronal differentiation may be under the control of canonical WNT-signaling, we used a transgenic mouse-line expressing *LacZ* under the influence of stable β-catenin. Here, we show that TH^+^ neurons of the midbrain receive canonical WNT-signaling during differentiation. Therefore, we suggest that early SHH-signaling is indirectly involved in mdDA development through early patterning of the midbrain area, whereas canonical WNT-signaling is directly involved in the differentiation of the mdDA neuronal population.

## Introduction

The mesodiencephalic dopaminergic (mdDA) group of neurons consists of different neuronal subsets, each dependent on a unique transcriptional code for their development [1]–[3]. These subsets are thought to be specified when terminal differentiation progresses [3]. Little is known about the early specification of the mdDA system, during which dopaminergic (DA) progenitors become post-mitotic and start to differentiate into immature mdDA neurons and later to fully differentiated mdDA neurons, expressing the rate-limiting enzyme for DA synthesis: tyrosine hydroxylase (TH). It is thought that Sonic Hedgehog (SHH), secreted by the floor plate (FP), is one of the early specification factors and plays a major role in the commitment of DA progenitors to the mdDA region [4].

SHH is a key regulator in many developmental processes and is expressed in at least three signaling centers in the developing embryo, the notochord, FP, and zone of polarizing activity [5]. Within the brain SHH is expressed in a gradient starting at the FP, which is required for correct dorsal-ventral (DV) patterning of the neural tube [6], [7]. This gradient is formed by SHH-N, the active component of SHH created by autoproteolytic cleavage [8], [9]. SHH-N acts as an antagonist of the Patched (PTCH1) receptor, thereby inducing FP and motor neuron development [9]. Binding of SHH-N to PTCH1 in the embryonic neural tube results in release of PTCH1-regulated inhibition of Smoothened (SMO) and regulation of the expression of SHH target-genes, like the glioblastoma (*Gli*) protein family members, *Gli1*, *Gli2*, and *Gli3* [5], [10]. The GLI-proteins on their turn are SHH-dependent regulators of SHH-targets, regulating gene expression under the influence of SHH [5]. The effect of SHH on the expression and activity of the GLI-proteins is different for each protein. SHH induces the activator GLI2A, and suppresses the processing of GLI3 into the repressor GLI3R [5], [10]. The precise function of GLI1 is not known, but its initial transcription is dependent on GLI2A-mediated SHH-signaling and is abolished when cells start expressing SHH themselves [11], [12].

The first indication that SHH might be involved in the development and differentiation of mice mdDA neurons came from the group of Rosenthal (1998). They have shown that DA neurons in cell culture develop at intersections between FGF8 and SHH expression, and suggested that these signaling molecules are both necessary and sufficient for the induction of DA neurons [7]. Earlier Hynes et al. (1995) indicated that SHH-N is involved in the induction of FP-cells, DA neurons, and motoneurons in the ventral neural tube of the rat [4]. Since SHH is expressed by FP-cells and DA neurons are thought to originate in both mice and rat near the midbrain FP, it has been speculated that SHH is critically involved in the differentiation of mdDA neurons [13]. To study the influence of SHH on mdDA development, Blaess et al. (2006) used a cre-lox system for *Smo*, one of the co-receptors for SHH signaling, with either engrailed-cre (*En*-cre) or nestin-cre. When SHH-signaling was abolished at embryonic stage (E) 9.0 (*Smo*-*En* conditional KO (cko)) the mdDA cell population is greatly reduced, but removal of *Shh* after E11.0 (Smo-Nestin cko) resulted in an almost normal development of mdDA cells. Therefore they suggest that SHH is crucial for the development of the mdDA system throughout the DV axis from E8.5 till E11.0 by regulating expansion via inhibition of cell death between E9.0 and E11.0. However, the *Gli2-En* cko showed barely any effect on the mdDA system, but did show a depletion of *Isl1* and *Nkx2.2* expressing motoneurons [14]. Furthermore, studies utilizing gene inducible fate mapping (GIFM) to mark the *Shh* and *Gli1* lineages at E7.5–E11.5 showed that in mdDA neurons *Gli1* and *Shh* have been expressed and thus might contribute to early mdDA cell differentiation [15]–[17]. On the other hand, some studies indicate that SHH does not promote a DA cell fate, but inhibits progenitors to acquire a DA cell-fate [18]–[20]. WNT-β-catenin signaling is thought to inhibit SHH in the ventral midline of the FP of the embryonic midbrain, allowing for neurogenesis and DA differentiation [19].

In order to gain more insight in the role of SHH-signaling during differentiation of mdDA neurons, we used a BAC-transgenic mouse model expressing enhanced green fluorescent protein (eGFP) under the influence of the *Gli1* promoter, a read-out for GLI2A-mediated SHH-signaling [11], [21]. This model can be used for the detection of SHH-signaling in TH expressing neurons of the midbrain. Since eGFP is stable for up to 2 days, this mouse model allows for the tracing of cells that have expressed *Gli1* at a specific point during embryonic development [22]. We examined *Gli1*-eGFP transgenic mouse embryos at E11.5, E12.5, and E13.5. Because the birth of mdDA neurons starts at E10.5 and peaks at E11–E12 [23], these time points provide a clear indication whether *Gli1* was expressed before (E9.5–E10.5) or during (E10.5–E13.5) mdDA differentiation in DA precursors and thus whether GLI2A-mediated SHH-signaling is involved in this process. Our data show that *Gli1*-eGFP is not induced in TH expressing mdDA neurons at all time points. However, *Gli1*-eGFP co-localizes with a subset of Brain-specific homeobox/POU domain protein 3A (BRN3A) expressing cells. Together, our data indicate that GLI2A-mediated SHH-signaling is not involved in mdDA differentiation, but rather in the specification of a subset of the red nucleus (RN). Because WNT-signaling has previously been suggested to play a role in mdDA neuronal differentiation [18]–[20] we determined whether differentiating mdDA neurons receive canonical WNT-signaling. Analysis of a transgenic mouse line expressing the *LacZ*-gene when stable β-catenin binds to the TCF/LEF binding-sites in the promoter shows clear co-localization between β-galactosidase and TH expressing neurons. We suggest that SHH-signaling is indirectly involved in mdDA neuronal differentiation through early patterning of the FP and midbrain area, whereas canonical WNT-signaling is directly involved in differentiation of the mdDA neuronal population.

## Materials and Methods

### Ethics Statement

All animal studies are performed in accordance with local animal welfare regulations, as this project has been approved by the animal experimental committee (Dier ethische commissie, Universiteit van Amsterdam and Universiteit Utrecht; DEC-UvA and DEC-UU), and international guidelines.

### Animals

The transgenic mouse line TG(Gli1-EGFP)DM197Gsat/Mmcd originate from the GENSAT Project at Rockefeller University and the transgenic mouse line B6.Cg-Tg(BAT-lacZ)3Picc/J (BAT-GAL) originate from the Jackson Laboratory. WT animals were derived from the C57BL/6 strain. Embryos were generated by crossing with C57BL/6 mice. Pregnant mice [embryonic day 0.5 (E0.5) is defined as the morning of plug formation] were killed by cervical dislocation. Embryos were collected in 1x PBS and immediately frozen on dry-ice, or fixed by immersion for 3–12 h in 4% paraformaldehyde (PFA) at 4°C. After PFA incubation, samples were washed in 1x PBS and cryoprotected O/N at 4°C in 30% sucrose. Embryos were frozen on dry-ice and stored at −80°C. Cryosections were cut at 16 µm, mounted on Superfrost Plus slides (Thermo Fisher Scientific), air-dried, and stored at −80°C until further use.

### Genotyping

#### TG(Gli1-EGFP)DM197Gsat/Mmcd strain

Genotyping of *Gli1*-eGFP transgenic embryos and mice was performed as follows:

100 ng of genomic DNA was used together with primer pair: FP -ccctcatccttccctgagac- and RP -tagcggctgaagcactgca-. The FP is positioned at the start of the *Gli1* sequence and the RP within the *Gfp* locus of the BAC-transgene. WT embryos show no product, whereas embryos containing the BAC-transgene show a product at 300 bp.

#### B6.Cg-Tg(BAT-lacZ)3Picc/J strain

Genotyping of the BAT-GAL transgenic embryos and mice was performed as follows:

100 ng of genomic DNA was used together with primer pair: FP -gttgcagtgcacggcagatacacttgctga- and RP -gccactggtgtgggccataattcaattcgc-. The FP and RP are both positioned in the *LacZ* sequence. Embryos carrying the *LacZ* gene show a product at 200 bp, whereas WT embryos show no product.

### Immunohistochemistry

Fluorescence immunohistochemistry was carried out as described previously [24], [25]. Briefly, cryosections were blocked with either 4% heat inactivated fetal calf serum (HIFCS) or 5% normal donkey serum (for sheep and goat primary antibodies) in 1x THZT and incubated with a primary antibody [Rb-TH (Pelfreeze, 1∶1000) [26], Sh-TH (Millipore AB1542, 1∶1000) [27], Rb-GFP (Abcam ab290, 1∶5000) [28], Rb-SHH (Santa Cruz sc-9024, 1∶400) [29], Ms-BRN3A (Santa Cruz sc-8429, 1∶200) [30], Gt-GLAST (Santa Cruz sc-7757, 1∶1000) [31], Rb-BG (Millipore AB986, 1∶1000) [32], Rb-WNT1 (Abcam ab15251, 1∶200) [33], Rb-LMX1A (kind gift of M. German, 1∶1000) [27]] diluted in 1x THZT O/N at 4°C. The next day sections were incubated with a secondary Alexafluor antibody (anti-rabbit, anti-sheep, anti-goat, anti-mouse) diluted 1∶1000 in 1x TBS for 2 h at RT. This procedure was repeated for double-labeling with a different primary antibody. After extensive washing in 1x PBS slides were embedded in Fluosave (Calbiogen) and analyzed with the use of a fluorescent microscope. Antibodies against SHH and BRN3A required antigen retrieval as follows. Slides were incubated with 0.1 M citrate buffer pH6 for 3 min at 800 W and 9 min at 400 W, cooled down to RT in a water bath, after which the protocol was followed as usual. Quantification of the BRN3A^+^ GFP^+^ population was performed as follows. BRN3A expressing cells in three coronal and sagittal sections were counted for stage E11.5 and E12.5 (N = 2 for each stage). Within this cell population the BRN3A-GFP double positive cells were counted separately, The relative amount of BRN3A^+^ GFP^+^ cells was calculated from these numbers and shown as percentage of the total BRN3A expressing population.

### *In situ* hybridization and combined TH-DAB IHC

*In situ* hybridization with digoxigenin (DIG)-labeled probes was performed as described previously [34]. Briefly, fresh frozen sections were fixed in 4% PFA for 30 minutes and acetylated with 0.25% acetic anhydride in 0.1 M triethanolamine for 10 minutes. Probe hybridization was carried out at 68°C O/N with a probe concentration of 0.4 ng/µl in a hybridization solution containing 50% deionized formamide, 5xSSC, 5x Denhardt's solution, 250 µg/mL tRNA Baker's yeast, and 500 µg/ml sonificated salmon sperm DNA. The following day slides were washed in 0.2x SSC for 2 hours at 68°C followed by blocking with 10% HIFCS in buffer 1 (100 mM TrisHCl, pH7,4 and 150 mM NaCl) for 1 hour at RT. DIG-labeled probes were detected by incubating with an alkaline-phosphatase-labeled anti-DIG antibody (Roche, Mannheim), using NBT/BCIP as a substrate.

DIG *in situ* hybridization was performed with the following probes: 510-bp mouse *Shh* fragment containing exon 1 [26], and *Wnt1*: bp 1205-2034 of mouse NM_021279.4.

After DIG *in situ* hybridization, sections were immuno-stained for TH. Slides were incubated in 0.3% H_2_O_2_ in Tris-buffered saline (TBS) for 30 minutes at RT. Thereafter, blocking was performed with 4% HIFCS in TBS. Slides were incubated O/N with primary antibody Rb-TH (Pelfreeze, 1∶1000) in TBS. The following day slides were incubated for 1 hour with goat-anti-rabbit biotinylated secondary antibody (Vector, 1∶1000) in TBS, followed by incubation with avidine-biotin-peroxidase reagents (ABC elite kit, Vector Laboratories 1∶1000) for 1 hour in TBS. The slides were stained with DAB (3,3'-diamino-benzidine) for a maximum of 10 minutes. Slides were dehydrated with ethanol and embedded with Entellan.

## Results

### During development TH^+^ neurons in the midbrain are positioned along- and perpendicular to radial glia in the mesodiencephalon

**Figure 1:**
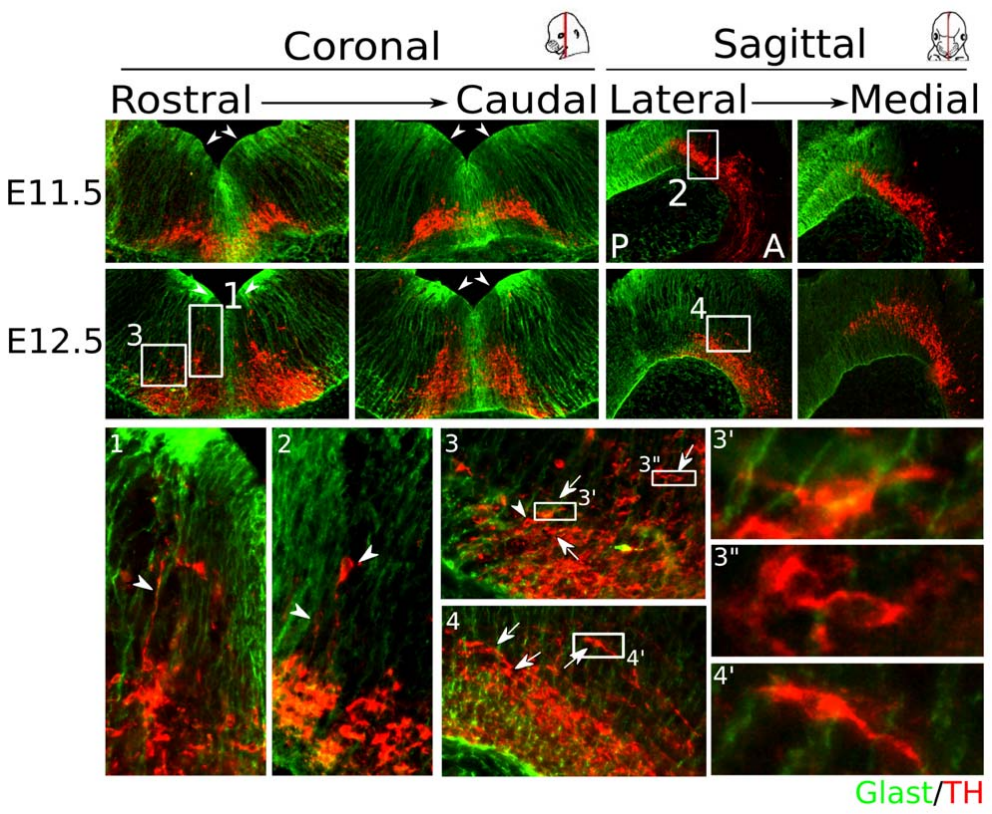
Cells of the mdDA system seem to originate in the FP and at the FP-BP boundary and are positioned along-side and perpendicular to radial glia. Radial positioned neurons can be detected from E11.5 onwards. TH^+^ neurons appear to originate in the FP and at the FP-BP boundary (white arrowheads in overview) and are positioned along-side radial glia (white arrowheads) (1–2). At E11.5 some radial positioned neurons can be detected rostrally, but most are present in the caudal part of the midbrain. When TH^+^ neurons reach the ventral part of the midbrain, most neurons are positioned tangential (white arrows), suggesting migration to more lateral and rostral regions (3–4 and 3′, 3", and 4′). A: Anterior; P: Posterior.

Although much is known about the development of the mesodiencephalic dopaminergic (mdDA) system at late stages, the question remains how these neurons differentiate in early stages and where mdDA neurons originate. It is has been described that the first mdDA neurons develop at embryonic day (E)10.5, but most neurons are born in between E11.0 and E12.0 [23]. In order to map the probable origin of mdDA neurons in the midbrain we performed fluorescent immunohistochemistry for the glutamate astrocyte-specific transporter (GLAST) and TH on WT embryos at E11.5 and E12.5. GLAST is a specific marker for radial glia allowing for the detection of mdDA neurons that are positioned along-side these cells (**Fig 1**). With LMX1A the location of the floor plate (FP) in the midbrain at E12.5 was determined (**Fig S1A**) [35], placing it in between the most dense areas of GLAST staining. Most mdDA neurons seem to originate within the FP and at the boundary with the basal plate (BP) in the caudal part of the midbrain, although some mdDA neurons appear to originate more rostral in the midbrain (**Fig S1B**). From the FP-BP boundary, mdDA neurons are positioned parallel to radial glia, suggesting radial migration from the FP to more ventral-medial parts of the midbrain (**Fig 1** **.1–2**). At the ventral-medial area TH^+^ neurons are oriented perpendicular to the radial glia, indicating that tangential migration takes place to more lateral and rostral parts of the mdDA region (**Fig 1** **.3–4** and **3′**, **3"**, and **4′**). At E11.5 most radial positioned mdDA neurons are observed in the medial part of the midbrain, and a few can be detected in more lateral regions which is not observed at E12.5. Since the mdDA neuronal field is still very small and compact, whereas the FP is broader at E11.5 than at E12.5 (**Fig 1**), it is possible that this causes the detection of radial positioned neurons at more lateral areas.

These findings support earlier data describing initial migration patterns of mdDA neurons [36], [37] and suggest that mdDA neurons originate within the FP and at the FP-BP boundary of the midbrain, after which they migrate to the diencephalic (P1–3) and midbrain domains.

### The SHH expression domain does not overlap with the majority of mdDA neurons during development

**Figure 2:**
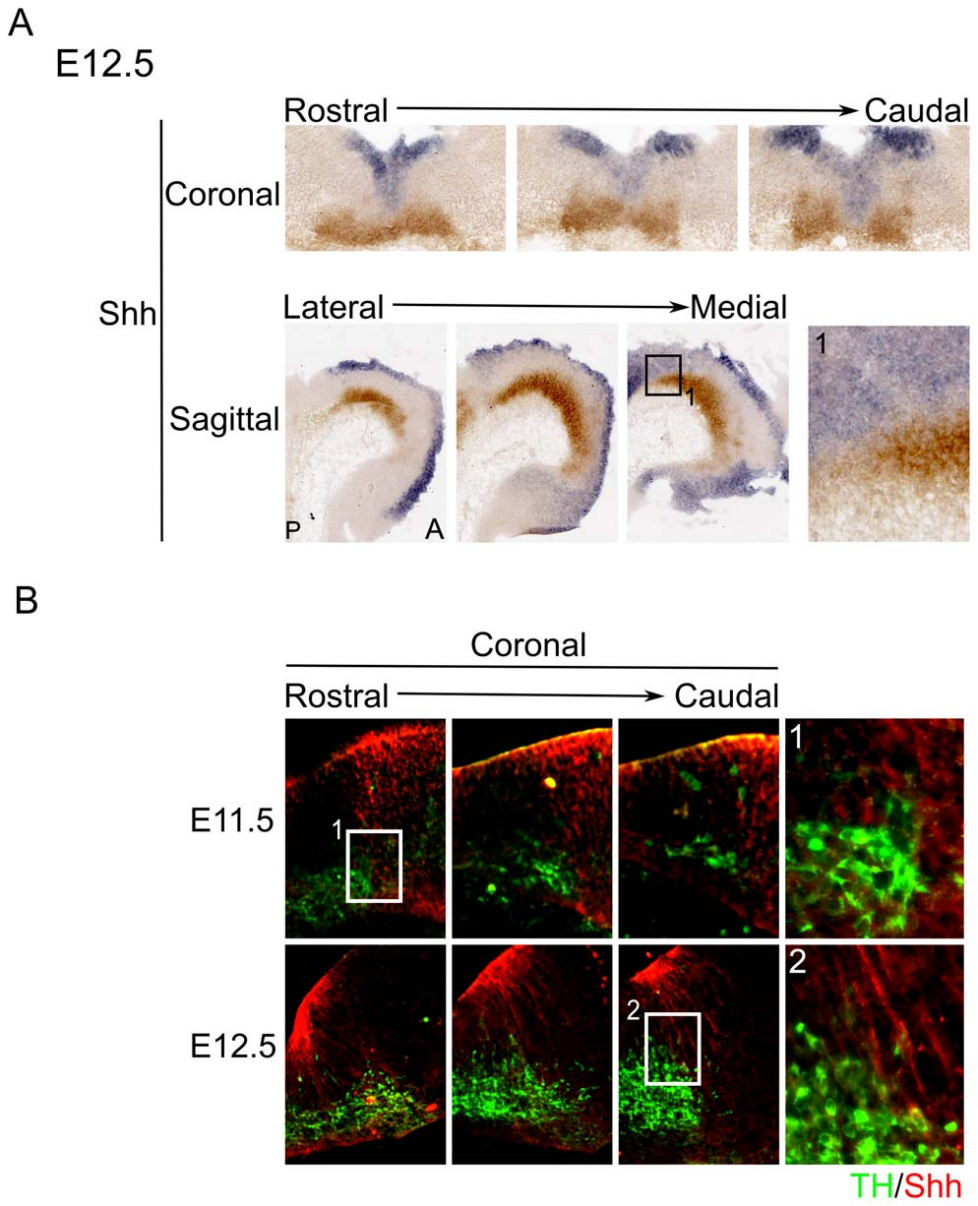
SHH expression does not overlap with the majority of the TH^+^ area of the midbrain. (**A**) *In situ* hybridization of *Shh* in comparison to TH-DAB shows expression in the ventricular zone at E12.5. However, expression of *Shh* is stronger in the BP than in the FP of the embryonic midbrain. *Shh* expression seems to end at the border with the TH expressing area (**1**). (**B**) At E11.5 and E12.5 SHH expression does not overlap with most of the TH^+^ cells in both rostral and caudal regions of the TH^+^ area. However, at the most lateral parts of the TH expressing area some overlap seems to exist with SHH (**1–2**).

Since SHH is known to be expressed in the FP of the developing embryo and migration data of mdDA neurons suggest that they originate in the midbrain FP, it is possible that mdDA neurons receive SHH signaling during differentiation, as previously suggested [15]–[17]. To determined whether mdDA neurons are born within the SHH expression area, we performed combined *in situ* hybridization for *Shh* and immunohistochemistry with DAB (3,3′-diamino-benzidine) for TH at E12.5 (**Fig2A**) and double fluorescent immunohistochemistry for SHH and TH at stage E11.5 and E12.5 (**Fig 2B**). Because most mdDA neurons are born around E11.0–E12.0 (see above), these stages will provide information whether mdDA neurons arise within the SHH expression domain. *In situ* data at E12.5 shows that *Shh* is expressed in the ventricular zone of the FP and BP, but a stronger expression is apparent in the BP (**Fig 2A**). However, *Shh* does not reach the TH^+^ neurons in the midbrain (**Fig 2A** **.1**). Double-labelling for SHH and TH at E11.5, when mdDA neuronal birth peaks, shows a medial gap in SHH expression in the midbrain, separating the SHH^+^ domain from TH expressing neurons (**Fig 2B** **.1**). During development the expression pattern of SHH changes. At E12.5 SHH is expressed in the rostral FP, directly above the TH^+^ neuronal population, but no SHH expression can be detected in the caudal FP, whereas it is present directly next to the TH expressing domain. These data show that most TH expressing neurons do not overlap with the SHH-gradient. Only some neurons at the lateral parts of the TH domain overlap with the SHH-gradient (**Fig 2B** **.2**). Positional data of mdDA neurons suggest that most TH neurons are born in the caudal FP and at the FP-BP boundary, indicating that they likely migrate in between the SHH expression domain towards their final positions. However, even though only a small lateral part of the TH domain overlap with the SHH-gradient, this does not indicate that TH expressing neurons do not receive Gli2A-mediated Shh signaling. Since very small levels of SHH can already induce the signaling cascade, TH^+^ neurons that do not show overlap with the SHH-gradient could still receive SHH-signaling by levels of SHH that are undetectable by means of fluorescent immunohistochemistry.

### *Gli1*-eGFP is present in the lateral-caudal midbrain, but not in mdDA neurons

**Figure 3:**
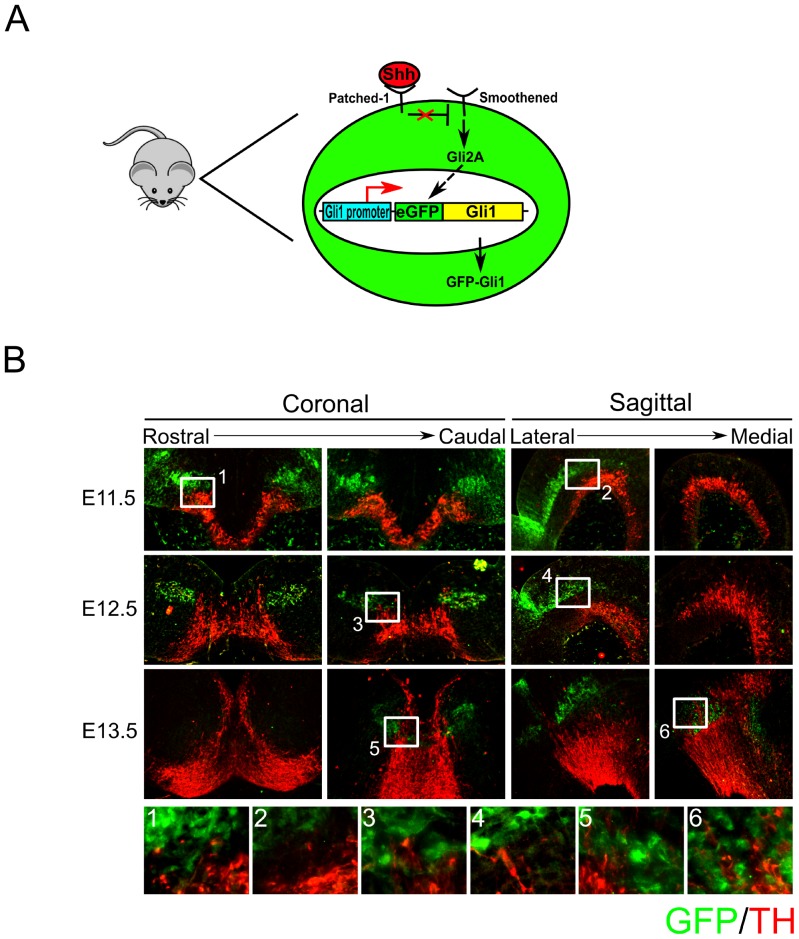
Expression of *Gli1*-eGFP and TH does not co-localize in the midbrain in the *Gli1*-eGFP-transgenic mouse line. (**A**) Model of the BAC-transgenic mouse line. This BAC-transgene contains several (minimum of 5) copies of eGFP which is transcribed under control of the *Gli1* promoter and immediately upstream of the *Gli1* sequence. When GLI1 expression is initiated by SHH-signaling, these cells start expressing eGFP. eGFP persists in the cell long after GLI1 has been broken down. Cells that expressed GLI1 can therefore be traced up to 2 days after expression of GLI1 has stopped. (**B**) Expression of *Gli1*-eGFP and TH does not co-localize at E11.5-E13.5 in the BAC-transgenic mouse-line. A strong separation between cells expressing *Gli1*-eGFP and TH^+^cells can be detected at all stages, but is most apparent at E11.5 and E12.5 (**1–4**). At E13.5 a few *Gli1*-eGFP expressing cells can be detected in the TH^+^ area. However, no co-localization can be detected (**5–6**). A: Anterior; P: Posterior.

In order to detect SHH-signaling in mdDA neurons, we used a *Gli1*-eGFP BAC-transgenic mouse line [21]. This mouse-line expresses eGFP under the control of the *Gli1* promoter (**Fig 3A**). As described previously, GLI1 can be used as a read-out for GLI2A-mediated SHH-signaling [11]. Since the stability of eGFP is higher than that of GLI1, we can trace SHH-signaling up to two days after GLI1 expression has ended [22]. Therefore, E11.5 embryos can provide information about GLI1 expression starting at E9.5, one day before mdDA neurons are born, until E11.5, when mdDA neuronal birth is at its peak [23]. To determine the spatiotemporal expression of *Gli1*-eGFP in the midbrain of the developing embryo, we performed fluorescent immunohistochemistry on *Gli1*-eGFP embryos at stage E11.5, E12.5, and E13.5 (**Fig 3B**). Coronal and sagittal sections show that *Gli1*-eGFP is expressed in two lateral domains, starting at the isthmus and progressing into the caudal region of the midbrain, not reaching the rostral parts. These domains move throughout development, starting at a ventral position at E11.5 and ending more dorsal, and even medially, in the midbrain at E13.5 (**Fig 3B**). Double-staining with TH and eGFP does not show co-localization between *Gli1*-eGFP and TH expression at any of the above mentioned stages (**Fig 3B**). At E11.5 and E12.5 the mdDA neuronal population and the *Gli1*-eGFP expressing population are strongly separated (**Fig 3B** **.1–4**). At E13.5 medial-caudal some *Gli1*-eGFP^+^ cells regionally overlap with TH expression. However, co-localization between the two proteins could not be detected (**Fig 3B** **.5–6**). In addition, double-labeling for AADC, another marker for mdDA neurons, also shows no co-localization with *Gli1*-GFP (data not shown)

Together, these results suggest that mdDA neurons do not receive GLI2A-mediated SHH-signaling during their differentiation. Above, we showed that TH^+^ neurons are likely to migrate in between the SHH-domain towards their final position. These data suggest that mdDA neurons would migrate in between the two *Gli1*-eGFP expression domains towards their final position.

### BRN3A expressing neurons of the red nucleus (RN) express GLI1 and are likely to receive GLI2A-mediated SHH-signaling during development

**Figure 4:**
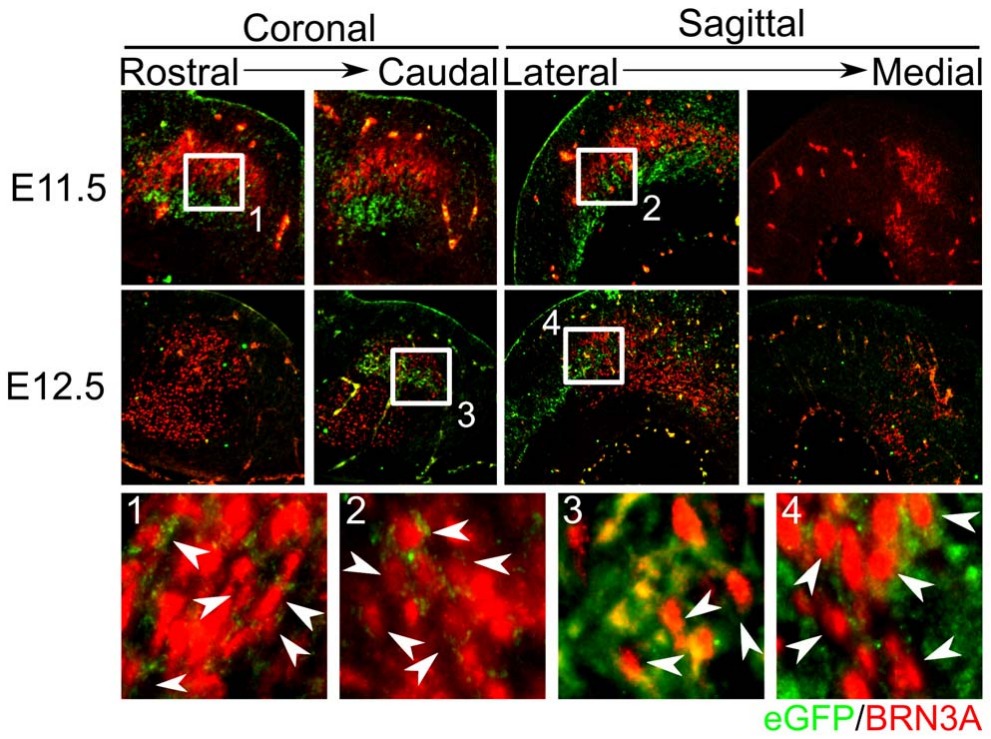
Expression of *Gli1*-eGFP co-localizes with BRN3A in the midbrain of *Gli1*-eGFP BAC transgenic mice. t E11.5, *Gli1*-eGFP expressing cells are present directly beneath the RN. Although most BRN3A expression and *Gli1*-eGFP are separated, some BRN3A^+^ cells co-localize with *Gli1*-eGFP expression (**1–2**). At E12.5 cells co-localization between nuclear BRN3A and cytosolic *Gli1*-eGFP in the caudal part of the RN is increased (**3–4**), indicating that (a subset of cells of) the RN probably develops under the control of GLI2A-mediated SHH-signaling. These cells typically start expressing BRN3A later in development, since at E11.5 only few *Gli1*-eGFP positive cells are detected in the RN. A: Anterior; P: Posterior.

We have shown that mdDA neurons do not express GLI1 and probably do not receive GLI2A-mediated SHH-signaling during their differentiation. Therefore, we investigated which group of neurons in the midbrain could be influenced by SHH-signaling. Double-labeling with BRN3A, a nuclear marker for RN neurons, allowed for the detection of *Gli1*-eGFP expression in neurons of the RN, which is involved in locomotion. We examined stage E11.5 and E12.5, indicating whether BRN3A^+^ neurons of the RN express GLI1 during their development and are therefore likely to receive GLI2A-mediated SHH-signaling between E9.5 and E12.5 (**Fig 4**). At E11.5 most BRN3A is expressed ventral from the *Gli1*-eGFP domains, but a small amount of cells is found to express both BRN3A in the nucleus and *Gli1*-eGFP in the cytosol within the caudal area of the midbrain (15.6% of the BRN3A expressing population) (**Fig 4** **.1** and **2**). At E12.5, the RN is expanded, but co-localization is still present in a dorsal-caudal population (9.4% of the BRN3A expressing population) (**Fig 4** **.3** and **4**). This suggests that GLI2A-dependent SHH-signaling is directly involved in the specification of a caudal subset of the RN between E9.5 and E12.5.

### Canonical WNT-signaling, and not SHH-signaling, is involved in mdDA neuronal differentiation

**Figure 5:**
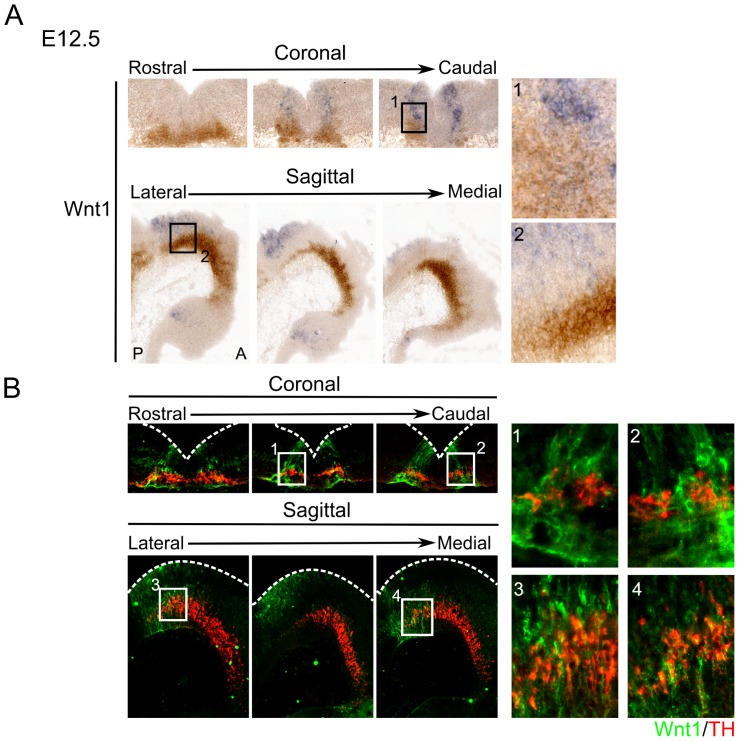
Expression of *Wnt1* mRNA and protein shows a clear overlap with TH^+^ neurons of the mdDA system. **A**) *In situ* hybridization of *Wnt1* in both coronal and sagittal sections shows overlap with TH expressing cells (**1–2**). (**B**) Immunostaining of WNT1 (green) and TH (red) at E12.5 midbrain coronal (**1–2**) and sagittal (**3–4**) sections shows a clear overlap between both proteins (**1–2**). Indicating that signaling via WNT1 could play a role in mdDA neuronal development. A: Anterior; P: Posterior.

Because GLI2A-mediated SHH-signaling is probably involved in the formation of the RN rather than the differentiating mdDA neurons, we aimed to investigate which early expressed signaling molecule could be involved in the differentiation of mdDA neurons. Several reports have suggested that WNT-signaling is critical for the development of the mdDA system [19], [38], [39]. The WNT-family is a large protein family, and although several members are known to be expressed in the mouse embryonic midbrain, not much is known about the function of these WNTs. However, WNT1 has been linked to the development of different subsets of the mdDA system and is a good possible candidate for the differentiation of mdDA neurons [40]–[43]. Combined *in situ* hybridization for *Wnt1* and immunohistochemistry for TH shows that *Wnt1* is expressed in two characteristic domains at the borders of the caudal FP, overlapping with TH expressing cells in the midbrain (**Fig 5A** **.1–2**). Immunohistochemistry of WNT1 and TH at E12.5 embryos shows a clear overlap between TH expressing neurons of the midbrain and WNT1 expression at this stage (**Fig 5B** **.1–2**). WNT1 is expressed in the FP and at the FP-BP boundary, previously pointed out as the possible place of birth of mdDA neurons, suggesting that WNT1 may be involved in the differentiation of mdDA neurons. As can be observed, the area in which the WNT1 protein is expressed reaches further rostral and ventral in the midbrain than the area in which the mRNA of *Wnt1* is expressed. This could be due to break-down of the transcript, while the protein is still present in the cell. Also, as WNT1 is a secreted factor, it is able to diffuse away from its place of synthesis, resulting in a larger WNT1 positive domain in the midbrain [44]. Although an overlap is observed between the two proteins, TH expressing neurons do not express WNT1 themselves. Since TH^+^ neurons are surrounded by WNT1 expressing cells, they are likely to receive canonical WNT-signaling during their development [45].

**Figure 6:**
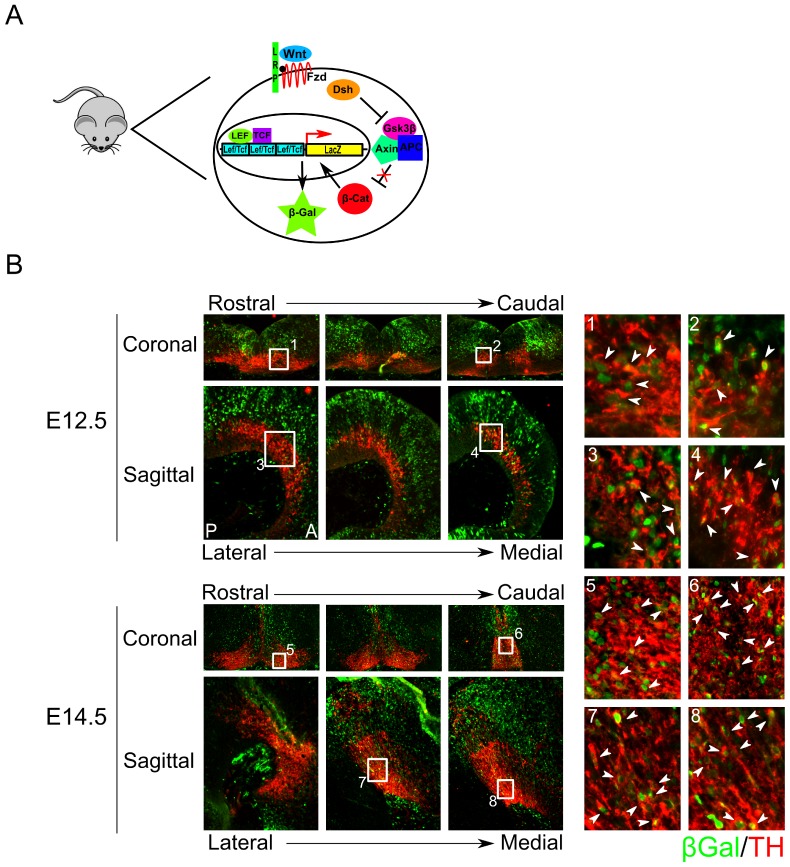
TH^+^ cells in the midbrain co-localize at both E12.5 and E14.5 with β-galactosidase, in a BATGAL expressing transgenic mouse line. A) Model of the transgenic BATGAL mouse line. β-galactosidase is expressed by binding of stable β-catenin to the TCF/LEF binding sites in the promoter of the *LacZ* gene. When β-catenin is activated, the *LacZ* gene in-cooperated in the genome is transcribed and β-galactosidase expressed. (B) At E12.5 β-galactosidase expression overlaps with TH both lateral and medial in the mdDA system (1–4). At E14.5 no β-galactosidase staining is detected lateral. However, medial many mdDA neurons can be detected to express β-galactosidase (5–8). Indicating that canonical WNT-signaling is or has been present in mdDA neurons. A: Anterior; P: Posterior.

In order to investigate whether TH^+^ neurons are subject to canonical WNT-signaling we made use of a reporter-model for canonical WNT-signaling (**Fig 6A**). This mouse model expresses β-galactosidase via binding of stable β-catenin to the TCF/LEF binding sites in the promoter of the in-cooperated *LacZ* gene. When cells receive canonical WNT-signaling at any given point during development, they will express β-galactosidase, which is present in the cell for a limited amount of time after canonical WNT-signaling has ended [46].

Fluorescent immunohistochemistry for β-galactosidase and TH at E12.5 and E14.5 revealed that mdDA neurons receive canonical WNT-signaling at these timepoints and likely during differentiation (**Fig 6B**). At E12.5 most co-localization between β-galactosidase and TH was detected in the medial part of the mdDA region (**Fig 6B** **.1–4**). However, some neurons in more lateral parts of the system are also β-galactosidase positive (**Fig 6B** **.3**). At E14.5, neurons located in lateral parts of the mdDA system do not express β-galactosidase, whereas the medial compartment shows almost complete co-localization between TH and β-galactosidase expressing neurons (**Fig 6B** **.5–8**). When **Fig 5B** and **Fig 6B** are compared, it is evident that not all canonical WNT-signaling in the midbrain overlaps with the expression of WNT1. Although it is likely that WNT1 plays a major role in this process, it is possible that other WNTs expressed in the midbrain induce canonical WNT-signaling and may contribute to the differentiation (of several subsets) of mdDA neurons. On the other hand, cells may have migrated away from the WNT1-source and stopped responding to WNT1 resulting in a loss of β-galactosidase expression in our used model system. Taken together, our data suggests that not SHH-signaling, but rather canonical WNT-signaling is involved in the differentiation of mdDA neurons.

## Discussion

The mesodiencephalic dopaminergic (mdDA) system is importantly involved in the regulation of associative motor learning, emotion, and reinforcement [47]. Although much research has been performed to the development and function of this system, still little is known about the early differentiation and origin of mdDA neurons. MdDA neurons are suggested to originate in the floor plate (FP) and basal plate (BP) of the midbrain [23]. From there they are thought to migrate radial along-side radial glia and to move in a tangential direction via nerve fibers expressing the cell-adhesion molecule L1 towards their final positions [36], [37], [48]–[50]. Our positional data of TH expressing neurons in comparison to radial glia supports this migration-model. Most tangential positioned neurons can be detected at E11.5, which suggests that the substantia nigra (SNc) develops before the ventral tegmental area (VTA), as shown by Bye et al. (2012) [51]. Because most mdDA neurons originate at the caudal FP and FP-BP boundary, differentiation of the mdDA neurons into the different subsets probably takes place later during development when the neurons are migrating towards or when they have reached their final position.

Several studies have suggested that SHH-signaling is involved in the development of mdDA neurons, but how this is regulated is not clear at present. To map GLI2A-mediated SHH-signaling both temporal and spatial in the embryonic mouse midbrain, we made use of a *Gli1*-eGFP transgenic mouse-line, expressing *Gli1*-eGFP after SHH-signaling [11]. Since eGFP is stable up to two days in the cell [22], we were able to detect which cells expressed GLI1 and are likely to have received GLI2A-mediated SHH-signaling between E9.5–E13.5, with mdDA neuronal birth peaking at stage E11–E12, by examining stage E11.5 until E13.5. It has been reported by Zervas et al. (2004) that GLI1 only contributes to some cells of the mdDA system before E7.5 [52]. More groups claim that mdDA neurons expressed GLI1 and SHH at some point during development using genetic inducible fate mapping (GIFM) systems for *Gli1* and *Shh* [15]–[17]. However, when data of these studies is examined closely, only few mdDA neurons can be detected to co-localize with GLI1 or SHH when tamoxifen is administered between E7.5 and E9.0 of development. Unlike these reports, we were not able to detect SHH or *Gli1*-eGFP in mdDA neurons, suggesting that they do not receive GLI2A-mediated SHH-signaling during differentiation. This does not rule out that any other possible modifiers of Gli1 activation may influence the end result as studied here.

When the position of the *Gli1*-eGFP expression domains and the individual cells within these domains is closely observed, it is apparent that the location of these domains changes through time (**Fig 3A**). At E11.5 the *Gli1*-eGFP domains are positioned ventrally in the midbrain, moving more dorsally from E12.5 to E13.5. Since *Gli1*-eGFP expressing neurons are positioned tangential, this movement is probably not caused by radial migration of these neurons. These domains likely move from ventral to dorsal because of expansion of the lower cell layers. The positional pattern of the TH^+^ neurons indicates that they originate at the FP and FP-BP boundary migrate in between and below the *Gli1*-eGFP expression domains to their final destination. Movement of mdDA neurons from the FP-BP to more ventral positions in the midbrain leads to expansion of the lower cell layers and pushes the *Gli1*-eGFP domains to a more dorsal location. Together, these results suggest that the midbrain area develops in an inside-out manner, similar as what is described for development of the cortex [53], [54].

Although we could not find *Gli1*-eGFP in mdDA neurons, we were able to detect *Gli1*-eGFP in BRN3A^+^ neurons from E11.5 onwards in a caudal subset of the red nucleus (RN). These results suggest that GLI2A-mediated SHH-signaling is involved in the specification of a subset of motor neurons in the RN, consistent with earlier reports [15], [17], which have shown with GIFM studies that SHH is expressed in BRN3A^+^ neurons from E9.5 onwards. Also, it has been shown by Blaess et al. (2006) that when GLI2A-mediated SHH-signaling is abolished, *Isl1* and *Nkx2.2* expressing motoneurons of the oculomotor nucleus are depleted, suggesting a role for SHH-signaling in the development of motor systems [14]. Because BRN3A^+^ cells express *Gli1*-eGFP at both E11.5 and E12.5 and co-localisation between these markers is even more evident at E12.5 it is possible that these cells are exposed to SHH-signaling before the onset of BRN3A expression. It is known that the RN contains several neuronal subsets [55], but how these subsets are specified during development is unclear. We suggest that the development of this specific subset is influenced by GLI2A-mediated SHH-signaling.

GLI2A-mediated SHH-signaling appears to be directly involved in the positioning and development of motoneurons in the spinal cord and in the oculomotor nucleus [11], [56]. However, although we could only detect *Gli1*-eGFP in neurons of the RN, many studies have pointed out that mdDA neuronal progenitors do receive SHH-signaling, mostly early in development [15]–[17]. Taken together, we suggest that GLI2A-mediated SHH-signaling is indirectly involved in the differentiation of mdDA neurons by means of early patterning and genesis of the midbrain area.

Since we could not confirm a role of GLI2A-mediated SHH-signaling in mdDA differentiation we investigated which signaling factor would be involved in this process. It has been shown that when canonical WNT-signaling is stabilized in the FP, SHH expression is abolished and TH^+^ neurons appear in the hindbrain, suggesting that WNT-signaling may be involved in mdDA neuronal development [38]. As suggested by several studies, the WNT-family member, which could be involved in the early differentiation of mdDA neurons is WNT1 [19], [40], [43], [57]. For instance, in *Wnt1* null-mutants, mdDA neurons do not differentiate properly [41] whereas a more caudal expression of WNT1 and stabilized β-catenin in the caudal FP results in an increase in caudal expression of TH, Nurr1, and Pitx3 [38], [41]. In this study we have shown that the expression of WNT1 overlaps with mdDA neurons and canonical WNT-signaling is present in TH expressing neurons of the midbrain. Most canonical WNT-signaling is detected in the medial TH^+^ region at both E12.5 and E14.5, although some signaling was detected more lateral at E12.5. The fact that most canonical WNT-signaling was detected in the medial part of the mdDA area could be explained in two ways. 1) Cells laterally positioned in the midbrain have received WNT1-signaling earlier during differentiation and migrated away from the medial-caudal part and the source of canonical WNT-signaling. Since mdDA neurons are thought to originate in the FP and at the FP-BP boundary [23], [50] and then migrate to more lateral and rostral positions, lateral TH^+^ neurons might have lost their responsiveness to canonical WNT-signaling. When these cells stop receiving canonical WNT-signaling they loose expression of β-galactosidase 2) Other signaling cascades, e.g., non-canonical WNT-signaling, are responsible for mdDA neuronal differentiation at other positions in the midbrain and canonical WNT-signaling is mainly necessary for the first step of differentiation and the development of the VTA. [23], [51] However, it is possible that canonical WNT-signaling in the midbrain area is induced by several WNTs and not solely by WNT1, since β-galactosidase was also apparent at areas where WNT1 is not expressed. Therefore we suggest that more WNTs may contribute to the (subset) specification of the mdDA system, although WNT1 probably plays a major part in this since most canonical WNT-signaling was detected in the WNT1 expression domain.

In conclusion, our results show that canonical WNT-signaling is likely involved in the differentiation of mdDA neurons, whereas GLI2A-mediated SHH-signaling is involved in the specification of a caudal subset of the RN. WNT-signaling mediated mdDA development is probably initiated by WNT1, as null-mutants for *Wnt1* show no differentiation of mdDA neurons and a more caudal expression of WNT1 leads to the development of TH^+^ neurons in the hindbrain [41]. Also, WNT1 is expressed in the caudal FP and at the FP-BP boundary, which is the area where TH^+^ neurons originate and canonical WNT-signaling is detected in mdDA neurons. Therefore, we suggest that early differentiation is under the influence of WNT1 canonical signaling in the midbrain caudal FP and FP-BP boundary.

## Supporting Information

Figure S1 **Expression of FP marker LMX1A in E12.5 coronal midbrain in comparison to Glast and TH.** (**A**) LMX1A is used as a marker for the FP and compared to the expression of GLAST and TH in E12.5 midbrain. LMX1A expression typically stops where GLAST expression is increased in the rostral and caudal midbrain (**1**). All TH^+^ neurons express LMX1A indicating that these neurons are probably derived from the FP and the FP-BP boundary (**2**). (**B**) TH-staining in E12.5 WT embryos shows radial positioned TH^+^ neurons in the medial part of the midbrain. Most radial positioned neurons are detected in the caudal part of the midbrain (**1**), whereas some can also be seen at more rostral parts (**2**). A: Anterior; P: Posterior.
(TIF)
