# Supplementary figures and images for: Mesodiencephalic Dopaminergic Neuronal Differentiation Does Not Involve GLI2A-Mediated SHH-Signaling and Is under the Direct Influence of Canonical WNT Signaling
Source: PLoS One. 2014 May 27;9(5):e97926. doi: 10.1371/journal.pone.0097926 (PMC4035267; doi:10.1371/journal.pone.0097926)

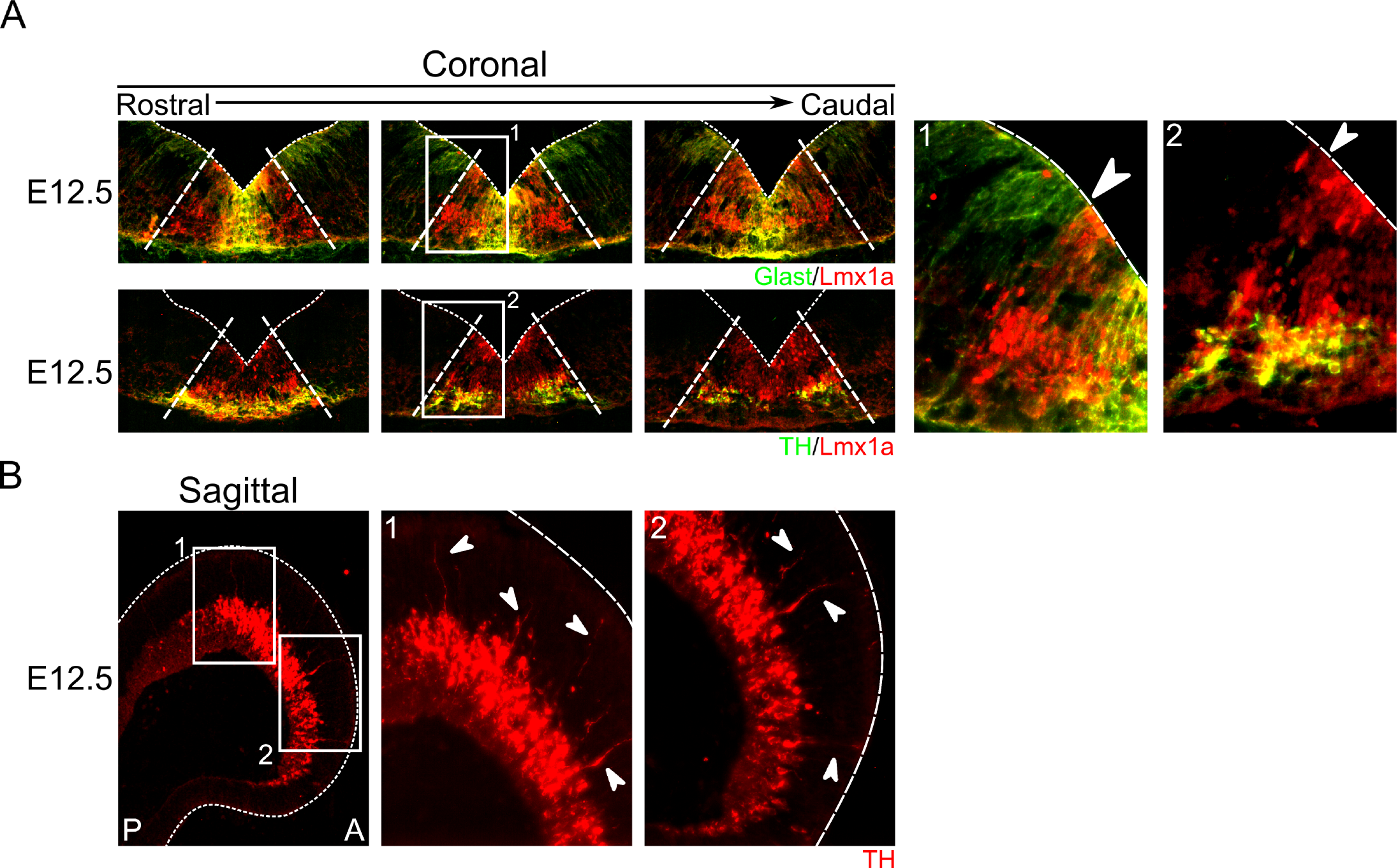

Supplement: Figure S1 — Expression of FP marker LMX1A in E12.5 coronal midbrain in comparison to Glast and TH. (A) LMX1A is used as a marker for the FP and compared to the expression of GLAST and TH in E12.5 midbrain. LMX1A expression typically stops where GLAST expression is increased in the rostral and caudal midbrain (1). All TH+ neurons express LMX1A indicating that these neurons are probably derived from the FP and the FP-BP boundary (2). (B) TH-staining in E12.5 WT embryos shows radial positioned TH+ neurons in the medial part of the midbrain. Most radial positioned neurons are detected in the caudal part of the midbrain (1), whereas some can also be seen at more rostral parts (2). A: Anterior; P: Posterior. (TIF) [file pone.0097926.s001.tif]
